# Understanding Cooperative Behavior Based on the Coevolution of Game Strategy and Link Weight

**DOI:** 10.1038/srep14783

**Published:** 2015-10-05

**Authors:** Keke Huang, Xiaoping Zheng, Zhijie Li, Yeqing Yang

**Affiliations:** 1 Department of Automation, Tsinghua University, Beijing 100084, China

## Abstract

In reality, the dependency relationship among individuals is heterogeneous and time-varying. Based on this fact, we present a new mechanism of coevolution of game strategy and link weight when analyzing the evolution of cooperation. In detail, we model the population on a regular network, on which the relationship between players is depicted by a weighted link, and prisoner’s dilemma has been applied to describe the interaction of players. Further, the impact of this mechanism on the cooperative behavior has been outlined. By conducting large-scale Monte Carlo simulations, we can easily draw a conclusion that this mechanism can promote cooperation efficiently. Compared with the traditional case, when the temptation of defection *b* is large, the fraction of cooperation is still able to keep in a high level. With a comprehensive examination of the distribution of stable link weight, it is evident that the coevolution mechanism would deviate the initial distribution. This mechanism induces the heterogeneity of players, which enhances the fraction of cooperation. Numerical simulations also indicate that an intermediate value of Δ/*δ* warrants an optimal resolution of prisoner’s dilemma. The mechanism of coevolution of game strategy and link weight has a practical significance and will provide new insight for the further research.

It’s generally acknowledged that networks are helpful in describing complex systems. Likewise, the interactions among people are often modeled within the network theoretical framework. Particularly interesting in this context is the time-varying nature of human interdependencies that qualitatively corresponds to the change of link weights in a network. Accordingly, we augment the standard mechanism of evolutionary game theory, whereby individuals adopt the game strategy of their more successful peers, with the simultaneous evolution of link weights. We thus examine the emergence of cooperative behavior as a result of the coevolution of game strategy and link weight.

How cooperative behavior evolved is still an open scientific question, generating humongous interest among scholars from multiple disciplines^1^,^2^,^3^,^4^,^5^. To address such an overarching question, the pioneering works in the field resorted to the framework of evolutionary game theory^6^,^7^,^8^. Prisoner’s dilemma^9^,^10^ and public goods game^11^,^12^,^13^,^14^ served as the paradigms for expressing a social dilemma in the cases of pairwise and group interactions, respectively. In these two types of games, defection always represents the optimal choice irrespective of the opponent’s decision. Yet, if defection is the preferred option, how did cooperation prevail in the real world? Following a number of attempts to resolve the conundrum of cooperation, Nowak in 2006 reviewed five rules for the promotion of cooperation named kin selection, direct reciprocity, indirect reciprocity, network reciprocity, and group selection^15^. Subsequently, the effects of a number of complementary mechanisms were also examined, including reward^11^,^13^,^16^, punishment^17^,^18^,^19^, and reputation^20^,^21^,^22^, to name a few. In parallel to the mechanisms that promote cooperation, the results of Nowak and May from 1992 sparked interest in the role of the spatial structure in the evolution of cooperative behavior^23^. Studies that followed placed evolutionary games into networks with small-world^24^,^25^,^26^, scale-free^27^,^28^,^29^, and a number of other topologies^30^,^31^,^32^,^33^,^34^,^35^. More recently, coevolutionary scenarios^36^ emerged as a promising way of improving the odds for the evolution of cooperation, whereby strategies and some other properties, such as updating rules or the topology of interactions, simultaneously evolve. Such setups affected the final network^37^, the fitness of players^38^,^39^, mobility^40^, or reputation^39^. Despite the progress so far, non-directional and unweighted networks received most of attention, whereas empirical studies indicated that the real-world systems are, by contrast, much more intricate. In fact, social networks are dynamic^41^ and often improperly represented if weights are ignored^37^,^42^. Therefore, the focus of future research may shift towards weighted networks.

We study the emergence of cooperative behavior in weighted, regular networks (i.e. lattices) due to the coevolution of game strategy and link weight. First, we place the population in a lattice in which the Prisoner’s Dilemma game is played among neighbors. Based on the payoffs from the game and the current state of link weights, each player gets certain utility. Second, the focal player reinforces (weakens) the links with neighbors who attracted above-average (below-average) payoffs. This is followed by a probabilistic update of the player’s strategy. Finally, using large scale Monte Carlo simulations, we study how the briefly described coevolutionary mechanism of strategies and link weights affects the level of cooperation.

## Results

**Figure 1:**
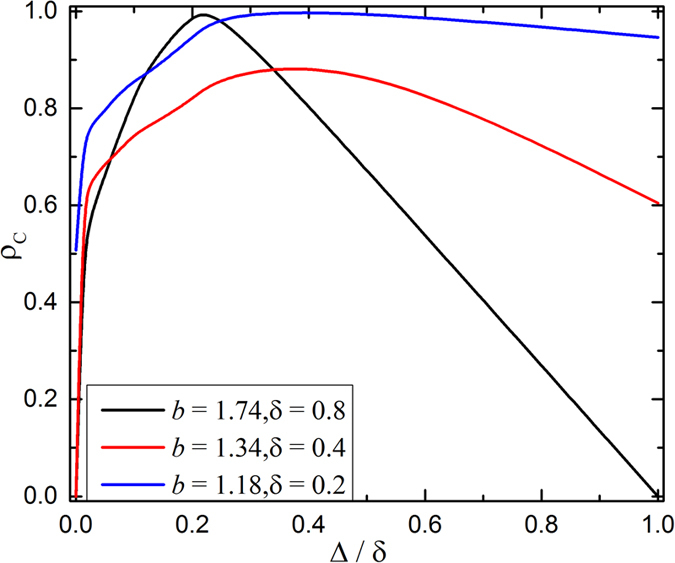
Relationship between fraction of cooperation *ρ*_*C*_ and Δ/*δ* for typical parameter of *b* and *δ*. It can be seen that an intermediate value of link weight evolution amplitude warrants an optimal resolution of social dilemmas. The size of the regular network is equal to 100 × 100.

We start by examining the influence of link weight coevolution on the sustainability of cooperation. Figure 1 shows how the fraction of cooperation *ρ*_*C*_ in dependence on link weight amplitude Δ/*δ* for fixed values of *b* and *δ*. It is clear that, irrespective of which case, the increment of Δ/*δ* first makes the fraction of cooperation *ρ*_*C*_ increase and then decline. In the interval Δ/*δ*∈[0.2, 0.5], the fraction of cooperation *ρ*_*C*_ reaches the peak, that is, an intermediate value of Δ/*δ* is most beneficial for the evolution of cooperation. Therefore, an intermediate value of link weight amplitude can optimally resolve the social dilemmas.

**Figure 2:**
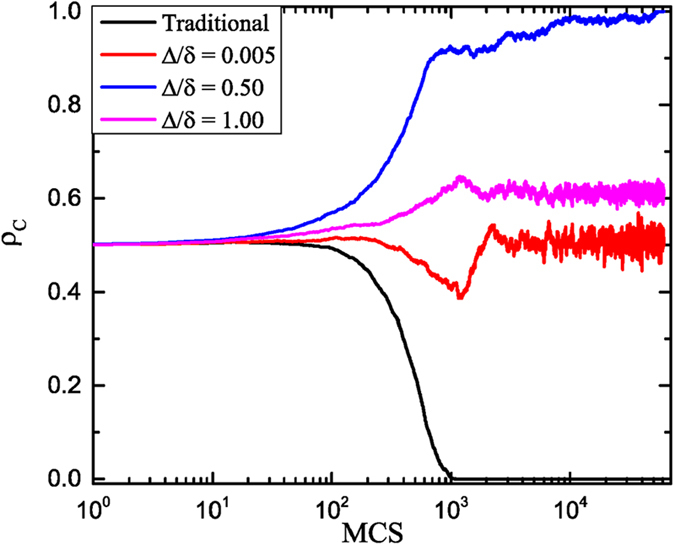
Time course of the fraction of cooperation on weight regular network. All results are obtained for*b* = 1.34, *δ* = 0.4. The black curve is corresponding to the traditional case (namely Δ = 0.0), the red curve is Δ = 0.1, the blue curve is Δ = 0.2 and the pink curve is Δ = 0.4. The size of the regular network is equal to 100 × 100.

**Figure 3:**
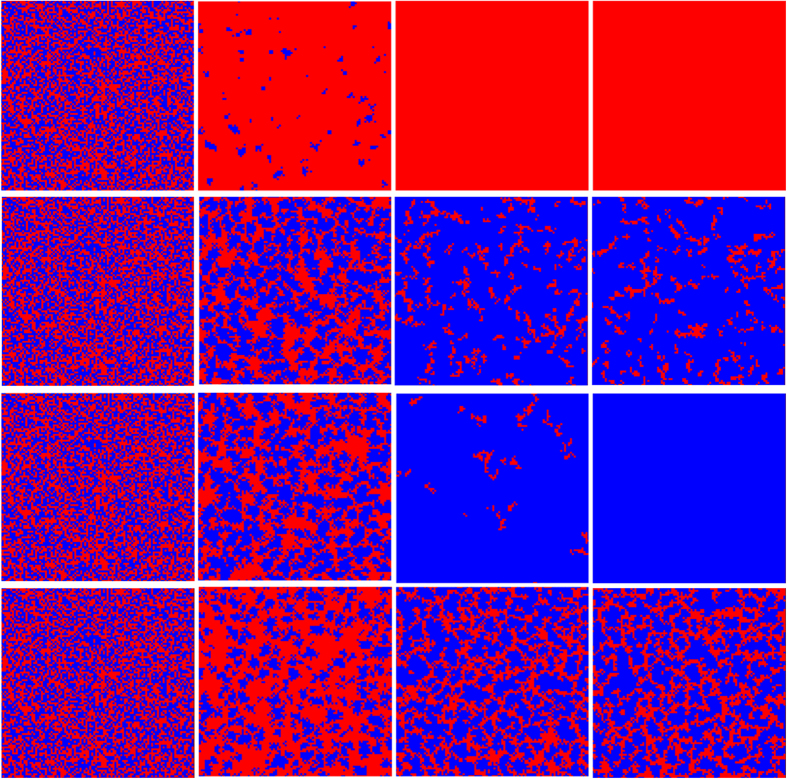
Typical snapshots of the distribution of strategy in step 0, 800, 1300 and 61000. All results are obtained for *b* = 1.34, *δ* = 0.4, and from the top to the bottom panel Δ/*δ* are equal to 0, 0.005, 0.5 and 1.0 respectively. In this Figure, the blue one represents cooperators, in contrast, the red one stands for defectors.

In networked population, the promotion of cooperation is termed as network reciprocity, which is usually denoted by the negative feedback mechanism of cooperation evolution process. In order to explain the optimal observation, it seems suitable to explore how this negative feedback mechanism is affected. Figure 2 features the time course of cooperative behavior for different values of link weight amplitude Δ/*δ*. To get more clear analysis, here we use the prepared initial state, where half is for cooperators, another half being for defectors, as demonstrated in Fig. 3. In Fig. 2, the black curve corresponds to the traditional case (namely Δ = 0.0), which vividly shows that the fraction of cooperation directly decreases till 0. That is to say, because of strong dilemma strength (i.e., large *b*), so-called negative feedback mechanism dies out in the traditional case. However, if we take the coevolution of game strategy and link weight into account, this mechanism comes back, even changes, and the fraction of cooperation is enhanced efficiently. Even for small Δ/*δ*, the distribution of link weight will become a little heterogeneous, which causes the fraction of cooperation decreasing in the first stages and then increasing, (namely, the negative feedback mechanism comes back). Subsequently, we investigate what happens for the curve of intermediate Δ/*δ* (i.e., Δ = 0.2) in the evolution processes, because this value guarantees the highest cooperation level in Fig. 1. Interestingly, cooperation will not shrink in the early stages but directly climb till reaching its complete dominance. This phenomenon can be attributed to change of the negative feedback mechanism, which is the key indication of cooperation explosion. Lastly, if Δ/*δ* is sufficiently large, the negative feedback mechanism changes to the adverse case, where cooperation firstly expands and then slightly declines to a middle level. Actually, for all these observations, they are closely related with the expansion of cooperation clusters, which will be explored in what follows.

In order to further validate the above explanation about the impact of the link weight on negative feedback mechanism, some typical snapshots of clusters evolution have been presented in Fig. 3. From the top to the bottom, the ratio of Δ/*δ* are equal to 0 (which is corresponding to the traditional case), 0.005, 0.5 and 1.0, and the time steps from the left to the right are *t* = 1, 800, 1300, 61000. With a fast scan of these snapshots, it is clear that the coevolution of game strategy and link weight can optimally resist the invasion of defectors with intermediate Δ/*δ*. For the traditional case, cooperation domain will be fast destroyed into some small cooperator clusters, which can not survive from the further exploitation. However, from the second panel, the clusters of cooperators survive from the initial attack, and then recover part of lost grounds. This is also the visual observation of negative feedback mechanism. As Δ/*δ* increases to middle level, the initial cooperators will fast expand into a giant cluster, and then reach the complete dominance, which indicates the extinction of negative feedback mechanism. However, when Δ is sufficiently large (lower panel), cooperation clusters will be divided into a few pieces, which can not guarantee the effective expansion and leads to middle cooperation level.

**Figure 4:**
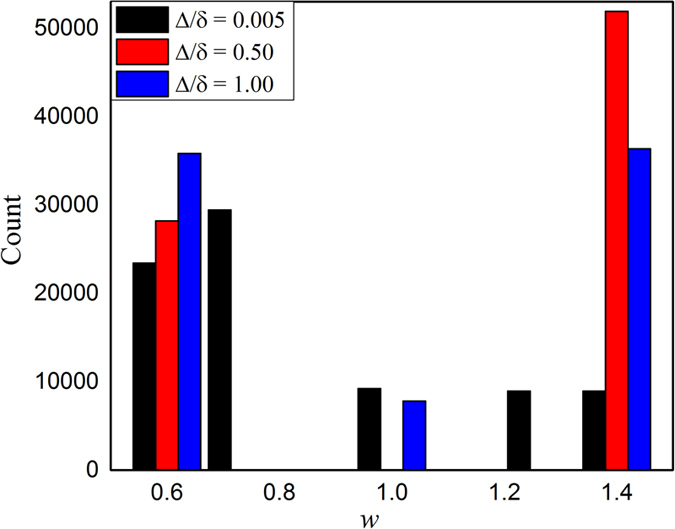
Stable distribution of link weight when *b* = 1.34, *δ* = 0.4. The size of the regular network is equal to 100 × 100.

Combining with the observations of Figs 2 and 3, it seems evident that the optimal promotion of cooperation is related with the changes of negative feedback mechanism in this coevolution scenario. For the sake of exploring the potential reason of these changes, Fig. 4 features the distribution of link weight for *b* = 1.34 and *δ* = 0.4 in stable state. It is clear that the link weight is not a single value no matter what value Δ is, namely, the coevolution of game strategy and link weight has introduced heterogeneous distributions of weight, which is usually a direct reason for the enhancement of cooperation. In particular, the variances of link weight of different Δ are equal to 0.7922, 1.3979 and 1.1496. Namely, when Δ = 0.2, the variance of the link weight distribution is the largest, which means the heterogeneity is the strongest in this case. The heterogeneity will, more or less, change the so called negative feedback mechanism and final cooperation level. Therefore, the coevolution of game strategy and link weight has great influence on the enhancement of cooperation and more heterogeneous weight distribution is more profitable for the evolution of cooperation. Here, it is worth mentioning that even if the initial distribution of link weight is heterogeneous, it does not influence the final equilibrium results and weight distribution after sufficient evolution time.

**Figure 5:**
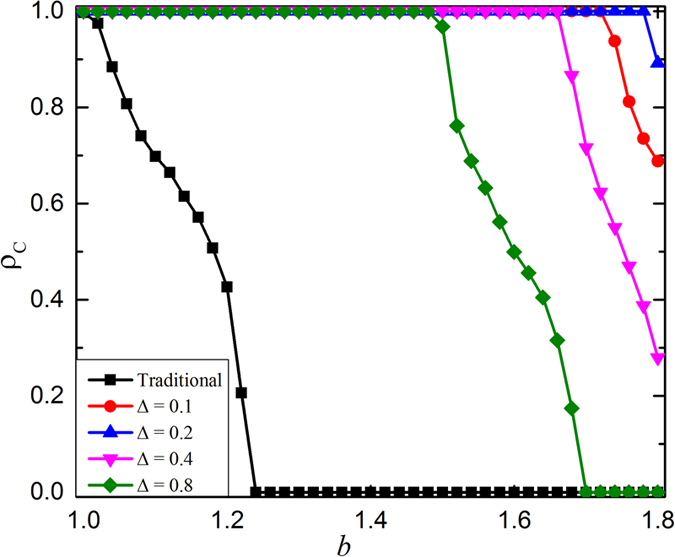
Relationship between the fraction of cooperation *ρ*_*C*_ and temptation parameter *b* when *δ* = 0.8 and Δ varied. Here, the black curve is corresponding to the traditional case (namely Δ = 0.0), the red curve is Δ = 0.1 , the blue curve is Δ = 0.2, the pink curve is Δ = 0.4 and the green curve is Δ = 0.8. The size of the regular network is equal to 100 × 100.

Now, let us turn our attention to the relationship between the fraction of cooperation *ρ*_*C*_ and temptation parameter *b* when the link weight is fixed in Fig. 5. In the traditional case, the fraction of cooperation *ρ*_*C*_ fast decreases with *b*, and will die out at around *b* = 1.24. However, if link weight is introduced, this case will greatly change. It is clear that even Δ = 0.1 can promote cooperation to complete dominance till sufficiently large *b*. With increment of Δ, this case will get further improvement. However, if Δ continues to increase, it will in turn impede the cooperation. For example, Δ = 0.8 can make cooperation go extinction again. Totally, this figure validates the observation of Fig. 1: middle link weight amplitude promotes cooperation best.

**Figure 6:**
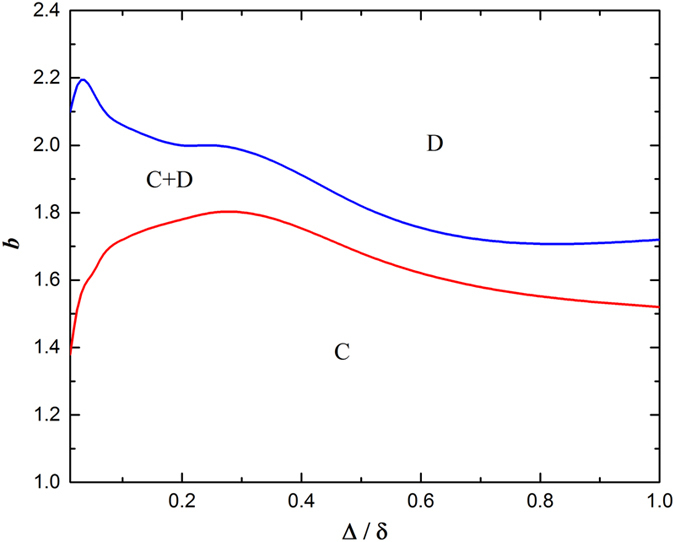
Typical phase diagram for the fraction cooperation *ρ*_*C*_ when Δ/*δ* and *b* varied. From this figure, it’s convincing to conclude that an intermediate value of Δ/*δ* is most profitable for the cooperators to survival and domination. The parameter δ = 0.8 and the size of the regular network is equal to 100 × 100.

Lastly, it is instructive to examine how this coevolution scenario affects the phase diagram in Δ/*δ*-*b* plane. In Fig. 6, blue curve corresponds to the boundary of the extinction of cooperators, while red curve is the boundary of the extinction of defectors. It is evident that with the increment of Δ/*δ*, both curves have a peak respectively, which means cooperation survives best at middle Δ/*δ*. For border line between *C* and *C+D* phases, this peak is broader, which means that middle Δ/*δ* can even guarantee the complete dominance of cooperation in higher *b*. With regard to the potential reason, it may be related with the change of interaction topology, which still needs further study in future.

## Methods

Here we consider the prisoner’s dilemma game, which is characterized by the temptation to defect *T* = *b*, reward for mutual cooperation *R* = *1*, and punishment *P* as well as the sucker’s payoff *S* equaling 0, whereby 1 < b < 2 ensures a proper payoff ranking. Initially each individual on player *x* is designated either as a cooperator (*s*_*x*_ = *C*) or defector (*s*_*x*_ = *D*) with equal probability. As the interaction, we use the regular square lattice (the size of lattice is equal to *N* = *L*^2^) with Moore neighborhood and periodic boundary conditions. Specially, link weight is introduced into evolutionary game via the adjacency matrix of network 
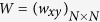
, which is symmetric for both ends of one link. For simplicity (yet without loss of generality), each edge linking node *x* and node *y* has the equal weight *w*_*xy*_ = 1 (before game), which however will adaptively change in accordance with the interaction.

The game is iterated forward in accordance with the Monte Carlo simulation procedure comprising the following elementary steps. First, a randomly selected player *x* acquires its payoff *P*_*xy*_ by playing the game with its neighbor *y*. Then, combing with edge weight *w*_*xy*_ and aforementioned payoff *P*_*xy*_, player *x* will get its accumulated utility as follows


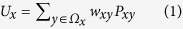


where Ω_*x*_ is the set of neighbors of player *x*. In particular, if the payoff *P*_*xy*_ of player *x* is larger than its average utility 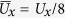 (i.e., 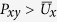), the link weight between *x* and *y* increases Δ as the reward (i.e., *w*_*xy*_ = *w*_*xy*_+Δ), otherwise decreases Δ as the punishment (i.e., *w*_*xy*_ = *w*_*xy*_−Δ ). As previous treatment^35^, we assume that the range of weight falls into 1 − *δ* ≤ *w*_*xy*_ ≤ 1 + *δ*_, where *δ*_ (_0_ ≤ *δ* ≤ 1) decides the heterogeneity of weight. When *δ* = 0 or Δ = 0, the weight of each link is equal to 1, which returns to the traditional case^23^,^28^. To be simple, we also use Δ/*δ*(*δ* ≠ 0) to denote link weight amplitude in the main text. Lastly, player *x* tries to update its strategy by picking up at random one neighbors *y* by comparing the respective utility *U*_*x*_ and *U*_*y*_. If *U*_*x*_ > *U*_*y*_, player *x* will keep its strategy for the next step. On the contrary, if *U*_*y*_ > *U*_*x*_, player *x* will copy the strategy of player *y* with a probability proportional to the utility difference


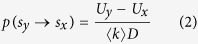


where *D* denotes the maximal possible payoff difference between both players *D* = *T*−*P* for the prisoner’s dilemma, 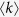 is the largest between the degree of player *x* and player *y*^28^. In one Monte Carlo step (MCS), each player is selected once on average to change its strategy.

The results of Monte Carlo simulations presented below were obtained on 100 × 100 lattices. The key quantity the fraction of cooperators *ρ*_*C*_ was determined within the last 1000 full MCS over the total 61000 steps. Moreover, since the coevolution may introduce additional disturbances, the final results were averaged over up to 10 independent realizations.

## Discussion

To conclude, we have studied the influence of coevolution setup of game strategy and link weight on the evolution of cooperation in prisoner’s dilemma game. It is found that intermediate link weight amplitude can provide best environment for the evolution of cooperation. Compared with the traditional case, the so called negative feedback mechanism is changed with the consideration of link weight. Further, the evolution trend is affected, which can be best reflected by the expansion of cooperation clusters. Middle link weight amplitude can make the existing clusters of cooperators expand into a giant cluster till its complete dominance. With regard to these observations, it is also related with the heterogeneous distribution of link weight. The stronger the heterogeneity is, the higher the final level of cooperation will be. Since link weight is ubiquitous in empirical networks, we hope that this work can shed some new lights on resolving the social dilemmas in realistic world.

## Additional Information

**How to cite this article**: Huang, K. *et al.* Understanding Cooperative Behavior Based on the Coevolution of Game Strategy and Link Weight. *Sci. Rep.* **5**, 14783; doi: 10.1038/srep14783 (2015).
